# 2-[4-(4,5-Dihydro-1*H*-pyrrol-2-yl)phen­yl]-4,5-dihydro-1*H*-imidazole

**DOI:** 10.1107/S160053680803818X

**Published:** 2008-11-22

**Authors:** Reza Kia, Hoong-Kun Fun, Hadi Kargar

**Affiliations:** a X-ray Crystallography Unit, School of Physics, Universiti Sains Malaysia, 11800 USM, Penang, Malaysia; b Department of Chemistry, School of Science, Payame Noor University (PNU), Ardakan, Yazd, Iran

## Abstract

The mol­ecule of the title compound, C_12_H_14_N_4_, lies about a crystallographic inversion centre. The five- and six-membered rings are twisted from each other, forming a dihedral angle of 18.06 (7)°. In the crystal structure, neighbouring mol­ecules are linked by inter­molecular N—H⋯N hydrogen bonds into one-dimensional infinite chains forming 18-membered rings with *R*_2_^2^(18) motifs. The crystal structure is further stabilized by weak inter­molecular π–π stacking [centroid–centroid distance = 3.8254 (6) Å] and C—H⋯π inter­actions.

## Related literature

For details of hydrogen-bond motifs, see: Bernstein *et al.* (1995 ▶). For a related structure and synthesis, see: Stibrany *et al.* (2004 ▶). For applications, see: Blancafort (1978 ▶); Chan (1993 ▶); Vizi (1986 ▶); Li *et al.* (1996 ▶); Ueno *et al.* (1995 ▶); Corey & Grogan (1999 ▶).
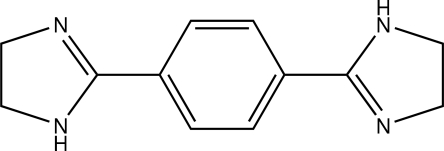


## Experimental

### 

#### Crystal data

C_12_H_14_N_4_
*M* *_r_* = 214.27
Triclinic, 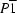

*a* = 4.8863 (2) Å
*b* = 5.1472 (2) Å
*c* = 10.2295 (4) Å
α = 104.414 (2)°
β = 93.885 (2)°
γ = 94.207 (2)°
*V* = 247.52 (2) Å^3^
*Z* = 1
Mo *K*α radiation
μ = 0.09 mm^−1^
*T* = 100.0 (1) K
0.56 × 0.17 × 0.15 mm

#### Data collection

Bruker SMART APEXII CCD area-detector diffractometer
Absorption correction: multi-scan (*SADABS*; Bruker, 2005 ▶) *T*_min_ = 0.950, *T*_max_ = 0.986
4616 measured reflections
1296 independent reflections
1208 reflections with *I* > 2σ(*I*)
*R*_int_ = 0.026

#### Refinement

*R*[*F* ^2^ > 2σ(*F* ^2^)] = 0.041
*wR*(*F* ^2^) = 0.116
*S* = 1.07
1296 reflections
101 parameters
All H-atom parameters refined
Δρ_max_ = 0.42 e Å^−3^
Δρ_min_ = −0.24 e Å^−3^

### 

Data collection: *APEX2* (Bruker, 2005 ▶); cell refinement: *APEX2*; data reduction: *SAINT* (Bruker, 2005 ▶); program(s) used to solve structure: *SHELXTL* (Sheldrick, 2008 ▶); program(s) used to refine structure: *SHELXTL*; molecular graphics: *SHELXTL*; software used to prepare material for publication: *SHELXTL* and *PLATON* (Spek, 2003 ▶).

## Supplementary Material

Crystal structure: contains datablocks global, I. DOI: 10.1107/S160053680803818X/tk2331sup1.cif

Structure factors: contains datablocks I. DOI: 10.1107/S160053680803818X/tk2331Isup2.hkl

Additional supplementary materials:  crystallographic information; 3D view; checkCIF report

## Figures and Tables

**Table 1:** Hydrogen-bond geometry (Å, °) *Cg*1 is the centroid of the N1/C1/C2/N2/C3 imidazoline ring.

*D*—H⋯*A*	*D*—H	H⋯*A*	*D*⋯*A*	*D*—H⋯*A*
N1—H1N1⋯N2^i^	0.87 (2)	2.18 (2)	3.0060 (13)	158.1 (15)
C2—H2*B*⋯*Cg*1^ii^	1.015 (15)	2.980 (15)	3.8882 (11)	149.6 (11)

Symmetry codes: (i) 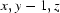; (ii) 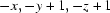.

**Fig. 1.**
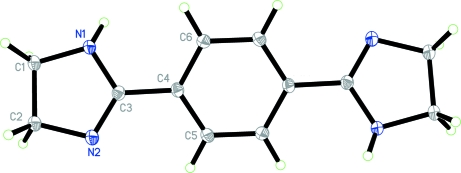
The molecular structure of (I) with atom labels and 50% probability ellipsoids for non-H atoms. Unlabelled atoms are related by -x + 1, -y, -z.

**Fig. 2.**
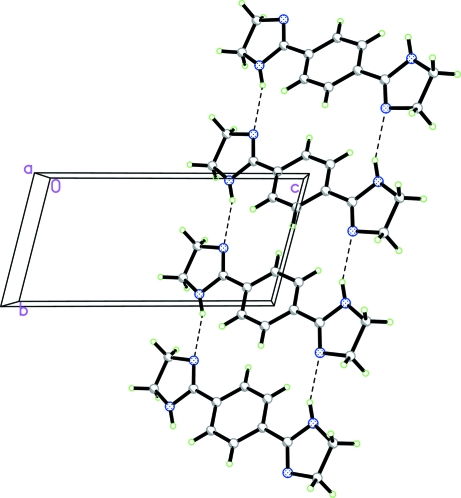
Partial crystal packing in (I), viewed down the a-axis showing one-dimensional infinite chains along the b-axis mediated by intermolecular N—H···N interactions (dashed lines).

## supplementary crystallographic information

### Comment

Imidazoline derivatives are of great importance because they exhibit significant biological and pharmacological activities including anti-hypertensive (Blancafort 1978), anti-hyperglycemic (Chan 1993), anti-depressive (Vizi 1986), anti-hypercholesterolemic (Li *et al.*, 1996) and anti-inflammatory (Ueno *et al.*, 1995) activities. These compounds are also used as catalysts and synthetic intermediates in some organic reactions (Corey & Grogan 1999). In consideration of the important applications of imidazolines, herein the crystal structure of the title compound, (I), is reported.

In compound (I), Fig. 1, bond lengths and angles are within the normal ranges and are comparable with a related structure (Stibrany *et al.*, 2004). The molecule lies about a crystallographic inversion centre. The five- and six-membered rings are twisted from each other, forming a dihedral angle of 18.06 (7)°. Intermolecular N—H···N hydrogen bonds form 18-membered rings producing *R_2_^2^(18)* ring motifs to link molecules into one-dimensional infinite chains along the *b*-axis, Table 1 and Fig. 2. The crystal structure is further stabilized by weak intermolecular π–π stacking [*Cg*1···*Cg*2^i^ = 3.8254 (6) Å; (i) 1 + *x*, *y*, *z*] and C—H···π (*Cg*1 and *Cg*2 are the centroids of the N1/C1/C2/N2/C3 imidazoline ring and the benzene ring, respectively) interactions, Table 1.

### Experimental

The synthetic method used for the preparation of (I) was based on previous work (Stibrany *et al.* 2004), except that 1,4-dicyanobenzene (10 mmol) and ethylenediamine (40 mmol) were used. Single crystals suitable for X-ray diffraction were obtained by evaporation of a methanol solution of (I) held at room temperature.

### Refinement

All hydrogen atoms were located from a difference Fourier map and refined freely: C—H ranged from 0.961 (16) to 1.015 (15) Å and N—H was 0.874 (18) Å.

### Crystal data

**Table:**

C_12_H_14_N_4_	*Z* = 1
*M**_r_* = 214.27	*F*_000_ = 114
Triclinic, *P*1	*D*_x_ = 1.437 Mg m^−^^3^
Hall symbol: -P 1	Melting point: 312 K
*a* = 4.8863 (2) Å	Mo *K*α radiation λ = 0.71073 Å
*b* = 5.1472 (2) Å	Cell parameters from 3789 reflections
*c* = 10.2295 (4) Å	θ = 2.5–30.3º
α = 104.414 (2)º	µ = 0.09 mm^−^^1^
β = 93.885 (2)º	*T* = 100.0 (1) K
γ = 94.207 (2)º	Block, colourless
*V* = 247.522 (17) Å^3^	0.56 × 0.17 × 0.15 mm

### Data collection

**Table:**

Bruker SMART APEXII CCD area-detector diffractometer	1296 independent reflections
Radiation source: fine-focus sealed tube	1208 reflections with *I* > 2σ(*I*)
Monochromator: graphite	*R*_int_ = 0.026
*T* = 100.0(1) K	θ_max_ = 29.0º
φ and ω scans	θ_min_ = 4.1º
Absorption correction: multi-scan(SADABS; Bruker, 2005)	*h* = −6→6
*T*_min_ = 0.950, *T*_max_ = 0.986	*k* = −6→6
4616 measured reflections	*l* = −13→13

### Refinement

**Table:**

Refinement on *F*^2^	Secondary atom site location: difference Fourier map
Least-squares matrix: full	Hydrogen site location: inferred from neighbouring sites
*R*[*F*^2^ > 2σ(*F*^2^)] = 0.041	All H-atom parameters refined
*wR*(*F*^2^) = 0.116	*w* = 1/[σ^2^(*F*_o_^2^) + (0.0717*P*)^2^ + 0.0714*P*] where *P* = (*F*_o_^2^ + 2*F*_c_^2^)/3
*S* = 1.07	(Δ/σ)_max_ < 0.001
1296 reflections	Δρ_max_ = 0.42 e Å^−^^3^
101 parameters	Δρ_min_ = −0.24 e Å^−^^3^
Primary atom site location: structure-invariant direct methods	Extinction correction: none

### Special details

**Table:**

Experimental. The low-temperature data was collected with the Oxford Cyrosystem Cobra low-temperature attachment.
Geometry. All e.s.d.'s (except the e.s.d. in the dihedral angle between two l.s. planes) are estimated using the full covariance matrix. The cell e.s.d.'s are taken into account individually in the estimation of e.s.d.'s in distances, angles and torsion angles; correlations between e.s.d.'s in cell parameters are only used when they are defined by crystal symmetry. An approximate (isotropic) treatment of cell e.s.d.'s is used for estimating e.s.d.'s involving l.s. planes.
Refinement. Refinement of *F*^2^ against ALL reflections. The weighted *R*-factor *wR* and goodness of fit *S* are based on *F*^2^, conventional *R*-factors *R* are based on *F*, with *F* set to zero for negative *F*^2^. The threshold expression of *F*^2^ > σ(*F*^2^) is used only for calculating *R*-factors(gt) *etc*. and is not relevant to the choice of reflections for refinement. *R*-factors based on *F*^2^ are statistically about twice as large as those based on *F*, and *R*- factors based on ALL data will be even larger.

### Fractional atomic coordinates and isotropic or equivalent isotropic displacement parameters (Å^2^)

**Table:**

	*x*	*y*	*z*	*U*_iso_*/*U*_eq_	
N1	0.99649 (17)	0.02202 (18)	0.30177 (9)	0.0140 (2)	
N2	1.05027 (18)	0.42785 (18)	0.25064 (9)	0.0161 (2)	
C1	1.2376 (2)	0.1595 (2)	0.39310 (10)	0.0148 (2)	
C2	1.2547 (2)	0.4433 (2)	0.36664 (10)	0.0158 (2)	
C3	0.92585 (19)	0.1869 (2)	0.21989 (10)	0.0124 (2)	
C4	0.70754 (19)	0.08912 (19)	0.10716 (10)	0.0124 (2)	
C5	0.6787 (2)	0.2263 (2)	0.00581 (10)	0.0138 (2)	
C6	0.5270 (2)	−0.1391 (2)	0.09997 (10)	0.0135 (2)	
H1A	1.402 (3)	0.062 (3)	0.3667 (15)	0.020 (3)*	
H1B	1.212 (3)	0.169 (3)	0.4906 (16)	0.024 (4)*	
H2A	1.443 (3)	0.498 (3)	0.3429 (16)	0.026 (4)*	
H2B	1.214 (3)	0.587 (3)	0.4488 (15)	0.020 (3)*	
H5	0.802 (3)	0.383 (3)	0.0096 (16)	0.025 (4)*	
H6	0.537 (3)	−0.239 (3)	0.1687 (15)	0.020 (3)*	
H1N1	0.989 (3)	−0.151 (4)	0.2656 (17)	0.029 (4)*	

### Atomic displacement parameters (Å^2^)

**Table:**

	*U*^11^	*U*^22^	*U*^33^	*U*^12^	*U*^13^	*U*^23^
N1	0.0135 (4)	0.0131 (4)	0.0147 (4)	−0.0009 (3)	−0.0033 (3)	0.0041 (3)
N2	0.0147 (4)	0.0150 (4)	0.0177 (5)	−0.0009 (3)	−0.0044 (3)	0.0047 (3)
C1	0.0125 (4)	0.0157 (5)	0.0153 (5)	−0.0008 (3)	−0.0030 (3)	0.0037 (4)
C2	0.0144 (5)	0.0153 (5)	0.0167 (5)	−0.0011 (4)	−0.0038 (3)	0.0041 (4)
C3	0.0102 (4)	0.0142 (5)	0.0130 (4)	0.0015 (3)	0.0006 (3)	0.0036 (3)
C4	0.0098 (4)	0.0134 (5)	0.0133 (4)	0.0009 (3)	0.0000 (3)	0.0025 (3)
C5	0.0109 (4)	0.0143 (5)	0.0159 (5)	−0.0009 (3)	0.0001 (3)	0.0041 (3)
C6	0.0128 (4)	0.0140 (5)	0.0142 (5)	0.0002 (3)	0.0003 (3)	0.0049 (3)

### Geometric parameters (Å, °)

**Table:**

N1—C3	1.3780 (13)	C2—H2B	1.015 (15)
N1—C1	1.4700 (12)	C3—C4	1.4787 (13)
N1—H1N1	0.874 (18)	C4—C5	1.3973 (14)
N2—C3	1.2944 (13)	C4—C6	1.4000 (14)
N2—C2	1.4808 (12)	C5—C6^i^	1.3881 (13)
C1—C2	1.5479 (14)	C5—H5	0.961 (16)
C1—H1A	0.997 (14)	C6—C5^i^	1.3881 (13)
C1—H1B	1.004 (16)	C6—H6	0.970 (15)
C2—H2A	1.006 (16)		
			
C3—N1—C1	107.38 (8)	C1—C2—H2B	112.1 (9)
C3—N1—H1N1	118.4 (11)	H2A—C2—H2B	106.8 (12)
C1—N1—H1N1	119.9 (10)	N2—C3—N1	116.89 (9)
C3—N2—C2	106.43 (8)	N2—C3—C4	123.28 (9)
N1—C1—C2	102.00 (8)	N1—C3—C4	119.77 (9)
N1—C1—H1A	108.9 (8)	C5—C4—C6	118.99 (9)
C2—C1—H1A	112.8 (9)	C5—C4—C3	119.82 (9)
N1—C1—H1B	112.3 (9)	C6—C4—C3	121.19 (9)
C2—C1—H1B	111.4 (9)	C6^i^—C5—C4	120.61 (9)
H1A—C1—H1B	109.3 (12)	C6^i^—C5—H5	119.7 (9)
N2—C2—C1	106.30 (8)	C4—C5—H5	119.6 (9)
N2—C2—H2A	109.2 (9)	C5^i^—C6—C4	120.40 (9)
C1—C2—H2A	111.9 (9)	C5^i^—C6—H6	117.6 (9)
N2—C2—H2B	110.6 (8)	C4—C6—H6	122.0 (9)
			
C3—N1—C1—C2	9.79 (10)	N1—C3—C4—C5	−165.14 (9)
C3—N2—C2—C1	3.06 (11)	N2—C3—C4—C6	−161.93 (10)
N1—C1—C2—N2	−7.84 (10)	N1—C3—C4—C6	15.11 (14)
C2—N2—C3—N1	3.71 (12)	C6—C4—C5—C6^i^	0.46 (16)
C2—N2—C3—C4	−179.17 (8)	C3—C4—C5—C6^i^	−179.29 (8)
C1—N1—C3—N2	−9.24 (12)	C5—C4—C6—C5^i^	−0.46 (16)
C1—N1—C3—C4	173.53 (8)	C3—C4—C6—C5^i^	179.29 (8)
N2—C3—C4—C5	17.82 (14)		

Symmetry codes: (i) −*x*+1, −*y*, −*z*.

### Hydrogen-bond geometry (Å, °)

**Table:**

*D*—H···*A*	*D*—H	H···*A*	*D*···*A*	*D*—H···*A*
N1—H1N1···N2^ii^	0.87 (2)	2.18 (2)	3.0060 (13)	158.1 (15)
C2—H2B···Cg1^iii^	1.015 (15)	2.980 (15)	3.8882 (11)	149.6 (11)

Symmetry codes: (ii) *x*, *y*−1, *z*; (iii) −*x*, −*y*+1, −*z*+1.

## References

[bb1] Bernstein, J., Davis, R. E., Shimoni, L. & Chang, N.-L. (1995). *Angew. Chem. Int. Ed. Engl.* **34**, 1555–1573.

[bb2] Blancafort, P. (1978). *Drugs Fut.* **3**, 592–592.

[bb3] Bruker (2005). *APEX2*, *SAINT* and *SADABS* Bruker AXS Inc., Madison, Wisconsin, USA.

[bb4] Chan, S. (1993). *Clin. Sci.* **85**, 671–677. 10.1042/cs0850671 8287658

[bb5] Corey, E. J. & Grogan, M. J. (1999). *Org. Lett.* **1**, 157–160. 10.1021/ol990623l 10822552

[bb6] Li, H. Y., Drummond, S., De Lucca, I. & Boswell, G. A. (1996). *Tetrahedron*, **52**, 11153–11162.

[bb7] Sheldrick, G. M. (2008). *Acta Cryst.* A**64**, 112–122. 10.1107/S0108767307043930 18156677

[bb8] Spek, A. L. (2003). *J. Appl. Cryst.* **36**, 7–13.

[bb9] Stibrany, R. T., Schugar, H. J. & Potenza, J. A. (2004). *Acta Cryst.* E**60**, o527–o529.

[bb10] Ueno, M., Imaizumi, K., Sugita, T., Takata, I. & Takeshita, M. (1995). *Int. J. Immunopharmacol.* **17**, 597–603. 10.1016/0192-0561(95)00057-9 8586488

[bb11] Vizi, E. S. (1986). *Med. Res. Rev.* **6**, 431–449. 10.1002/med.2610060403 2877125

