# 2-[4-(4,5-Dihydro-1*H*-pyrrol-2-yl)phen­yl]-4,5-dihydro-1*H*-imidazole. Corrigendum

**DOI:** 10.1107/S1600536809023320

**Published:** 2009-06-27

**Authors:** Reza Kia, Hoong-Kun Fun, Hadi Kargar

**Affiliations:** a X-ray Crystallography Unit, School of Physics, Universiti Sains Malaysia, 11800 USM, Penang, Malaysia; b Department of Chemistry, School of Science, Payame Noor University (PNU), Ardakan, Yazd, Iran

## Abstract

The chemical name of the title compound in the paper by Kia, Fun & Kargar [*Acta Cryst.* (2009), E**64**, o2406] is corrected.

## Abstract 2

Corrigendum to *Acta Cryst.* (2009), E**64**, o2406.

In the paper by Kia, Fun & Kargar [ *Acta Cryst.* (2009), E**64**, o2406], the chemical name given in the *Title* should be ‘2,2′-(*p*-phenylene)bis(4,5-dihydro-1*H*-imidazole)’.

